# Simple action alters attention towards visual features

**DOI:** 10.3758/s13414-021-02259-4

**Published:** 2021-03-08

**Authors:** Zixuan Wang, Blaire J. Weidler, Pei Sun, Richard A. Abrams

**Affiliations:** 1 Department of Psychology, Tsinghua University, Haidian, Beijing, China; 2 Department of Psychology, University of California, Berkeley, CA USA; 3 Department of Psychology, Towson University, Towson, MD USA; 4 Department of Psychological and Brain Sciences, Washington University, St. Louis, MO USA

**Keywords:** Action, Attention, Visual search

## Abstract

Recent studies have revealed an *action effect*, in which a simple action towards a prime stimulus biases attention in a subsequent visual search in favor of objects that match the prime. However, to date the majority of research on the phenomenon has studied search elements that are exact matches to the prime, and that vary only on the dimension of color, making it unclear how general the phenomenon is. Here, across a series of experiments, we show that action can also prioritize objects that match the shape of the prime. Additionally, action can prioritize attention to objects that match only one of either the color or the shape of the prime, suggesting that action enhances individual visual features present in the acted-on objects. The pattern of results suggests that the effect may be stronger for color matches – prioritization for shape only occurred when attention was not drawn to the color of the prime, whereas prioritization for color occurred regardless. Taken together, the results reveal that a prior action can exert a strong influence on subsequent attention towards features of the acted-on object.

## Introduction

The ongoing interactions between action and perception play a central role in the control of behavior. While there is a long tradition of research that reveals the importance of visual information in the control of ongoing movement (e.g., Woodworth, 1899), recent research has revealed, conversely, that the way in which individuals interact with the environment can affect how they see. For example, people often adopt an action-centered perceptual representation when they are interacting with objects (Bloesch, Davoli, & Abrams, 2013; Tipper, 1992). And preparing a specific action facilitates perception of features relevant to that action (e.g., Wykowska, Schubö, & Hommel, 2009). Additionally, if people can interact with an object through a reach-extending tool they perceive that object as closer – presumably because their capabilities to interact with the object have changed (e.g., Abrams & Weidler, 2015; Suh & Abrams, 2018a; Witt, Proffitt, & Epstein, 2005). Even the position of an individual’s hands relative to nearby objects can influence perception (e.g., Abrams, Davoli, Du, Knapp, & Paull, 2008; Abrams & Weidler, 2014; Thomas, 2015).

The close connections between action and perception systems seem even more remarkable in light of the well-known observations that vision in support of action is at least somewhat segregated from visual systems mediating other aspects of cognition and perception (e.g., Goodale & Milner, 1992; Mishkin, Ungerleider, & Macko, 1983). In the present paper we focus on a recently discovered influence of action on perception. In particular, several studies have shown that performing a simple action can affect subsequent visual perception and attention. Buttaccio and Hahn (2011) first showed that simply responding to an object can prioritize features of that object in the future. On each trial of their experiments, participants first saw a pre-cue (e.g., a color word like “blue”) followed by a colored shape (the prime). Then, based on the match between the cue and the word (e.g., if the shape was blue), they sometimes made an action (a key press) when the prime was present. Next, participants searched an array for a tilted line and indicated its tilt. Importantly, the lines were embedded in colored shapes, and the color of the prime was always present in the search display. On valid trials, the tilted line target was in the prime’s color whereas on invalid trials the prime’s color contained an untilted distractor line.

Buttaccio and Hahn (2011) found that the location of the prime’s color relative to the target (i.e., it’s validity – whether it contained the target or a distractor) had a profound effect on search performance – but only following an action. When participants had not responded to the prime, validity did not affect search performance. Thus, the authors argued that the simple action of pressing a key had somehow enhanced the prime’s color, so that it received priority in the visual search task. Subsequent research on this finding, *the action effect,* has further established the robustness of the finding by revealing that even when participants are pre-cued to respond prior to the prime’s onset and need not focus on any of its features, action still affects later perception (Weidler & Abrams, 2014). Furthermore, the effects of action can compete with bottom-up salience – targets containing acted-on features still receive priority even if the target is a uniquely salient element (e.g., a color or size singleton; Weidler & Abrams, 2016). Additionally, recent studies have shown that an action can affect eye movements during the search (Wang, Sun, Sun, Weidler, & Abrams, 2017; Weidler, Suh, & Abrams, 2018), and can even bias attention toward the color of primes that were not consciously perceived (Suh & Abrams, 2018b).

The action effect reveals a pervasive aftereffect of simple action and could serve an important role in guiding ongoing behavior. In particular, a bias toward features of acted on objects could facilitate repeated actions to the same object. Thus, behaviors such as tool use – in which repeated actions are often made to a single object – or foraging, in which successive actions are made to similar-looking objects – might be facilitated. But many basic questions about the action effect remain unanswered. In particular, it currently remains unclear whether action can prioritize all features of an object. For example, when serving as a foraging facilitator, do actions bias attention only toward objects that match the color of the desired fruit, or does shape and size matter also?

The gap in our present knowledge about the action effect stems from two major limitations of the previous research. First, all past research focusing on the action effect, with only one exception, has tested only whether the color of an object can be prioritized by action (the sole exception did not examine visual features at all, but instead studied semantic relations; Weidler & Abrams, 2018). There are some reasons to expect that color may have a special status compared to other basic features, perhaps because it provides salient cues for distinguishing objects from one another (Goolsby & Suzuki, 2001). Also, in a different paradigm that examines the effects of past events on visual search (*priming of pop-out*), color often has the most robust and reliable effects (e.g., McBride, Leonards, & Gilchrist, 2009; Kristjánsson, 2006).

Despite the special status of color, shape is also an important attribute of objects, and there is some reason to suspect that action could enhance attention to shape. In particular, previous research on effects of action *preparation* suggests that attention to features other than color is closely tied to the actions being made. For example, when people prepare to make a grasping movement, more attention is devoted to object size than when they prepare to make a poking action (e.g., Wykowska et al., 2009). Additionally, when a grasping movement is prepared to a target with a specific color and orientation, people are less likely to erroneously look to a distractor with a mismatching orientation, but no less likely to look to a mismatching color, compared to when pointing movements are planned (Bekkering & Neggers, 2002). Thus, despite the salience of color, features such as shape and orientation may be particularly emphasized when action is relevant. While these situations differ in some ways from the conditions under which the action effect has been studied, they serve to reveal the extensive interconnections between action and perception systems. Given these findings it seems quite possible that features other than color will support an action effect. Thus, one of our goals in the present study was to determine whether action is capable of influencing subsequent perception of objects that match a feature of the acted-on object other than color (and in this case, we chose shape).

The second limitation of the existing action effect research is that in most previous studies the identical prime object would appear in both the prime task and the search task. This makes it impossible to disentangle whether action enhances attention to whole objects (e.g., a blue circle) or simply to the individual component visual features (e.g., the color blue and the shape circle). The sole exception comes from Buttaccio and Hahn’s (2011) Experiment 3 in which participants made an action if the shape of the prime matched a previously cued shape name. Then in the subsequent search task, only the color of the prime (but not the shape) appeared. They found an action effect for color in that experiment, suggesting that action might be able to prioritize individual features of the prime. The finding, while important, is limited only to the feature of color, and has yet to be replicated. Thus, the second goal of the present set of experiments was to learn more about whether an action can prioritize individual features of the acted-on object.

Some previous explanations for the action effect permit some speculation about whether individual visual features will be prioritized. In particular, Huffman and Pratt (2017) suggested that the action effect occurs because the action biases competition in the perceptual system in favor of features of the prime in a manner similar to the biased competition that has been shown to occur for stimulus features that match those of a sought-for target (e.g., Bichot, Rossi, & Desimone, 2005; Desimone & Duncan, 1995). If this is the case then it might be expected that individual features of the prime would indeed lead to an action effect, since the biased competition has been shown to occur in brain areas responsive to basic features such as color and orientation (Reynolds, Chelazzi, & Desimone, 1999), or motion (Recanzone, Wurtz, & Schwarz, 1997). On the other hand, Weidler and Abrams (2018) showed that an action toward a word prime facilitated search for the object depicted by the word. Such an effect could not stem from enhancement of perceptual features, but instead must operate at a semantic level. Thus, those results suggest the possibility that the benefits of an action might occur only at a high level, encompassing the entire prime, and not be triggered by individual perceptual features of the prime.

### Feature-based attention

Addressing the questions we have identified about the action effect may also inform, and be informed by, an ongoing debate regarding the existence of *exogenous feature-based attention*. Some researchers have shown that it might be possible for people to reflexively prioritize individual features of a primed shape. For example, Lin, Hubert-Wallander, Murray, and Boynton (2011) reported that participants found a target shape more quickly if it matched the color of a previously presented (and uninformative) prime object, even when the shape and location of the prime and target did not match. Similar results have been reported by Huang et al. (2018). The action effect paradigm presents participants with a very similar situation (an uninformative colored shape presented at a non-target location), and these earlier findings would suggest that acting upon an object might indeed lead to prioritization of the object’s color, even if other features of the target (such as shape) do not match the prime. However, other researchers more recently have failed to find evidence of the existence of exogenous feature-based attention (Donovan, Zhou, & Carrasco, 2020). More work on the action effect, such as that reported here, may help to reveal more about the precise conditions under which exogenous feature-based attention can occur.

### Theory of event coding

The present investigation is also relevant to work on *event files* – episodic representations of recently produced responses and features of the stimuli to which they were directed. In many of the studies examining these representations, participants initially make an arbitrary response (a simple keypress) to a visual stimulus – much like what happens in action effect experiments when the prime appears. Next, the participant is required to detect or discriminate a subsequent stimulus and to again produce a response. An important finding from this research is that features of the initial stimulus appear to become bound with the initial response – as evidenced by response latencies to the second stimulus, which tend to be slower if either the earlier response must be paired with a new visual feature, or if a previously seen feature must now be paired with a new response (e.g., Hommel, 1998, 2004). Of relevance to the present investigation, it has been shown that individual features are bound to responses – not conjunctions of several features (e.g., Hommel & Colzato, 2004; Hommel, 2007). If the action effect relies on some of the same mechanisms that support event files, as has been suggested by some researchers (e.g., Weidler & Abrams, 2014), then it might be expected that individual featural overlap would be sufficient to yield an action effect – the prime need not be identical to the target as has been the case in most of the studies to date.

Additionally, some work on event files suggests that features other than color might indeed yield an action effect. Singh, Moeller, Koch, and Frings (2018) showed that even irrelevant features can be bound into an event file (if they had been attended). Of note, the features they studied included the affective valence of a word, suggesting that the binding is not limited to perceptual features. This raises the possibility that features other than color could also facilitate an action effect, assuming that the event file results are relevant to the action effect phenomenon.

To investigate the questions above, in the current study, Experiments 1a and 1b address whether simple action can modify subsequent attention towards the shape of an acted-on object. In Experiments 2a–2c, we study the extent to which action can enhance attention towards individual object features such as color or shape separately.

## Experiments 1a and 1b

The goal of Experiments 1a and 1b was to determine whether action can modify subsequent shape perception. Thus, all stimuli here were the same color, and varied only in their shape (simple shapes in Experiment 1a and complex shapes in Experiment 1b). If action affects subsequent shape perception, then after an action to the prime, search reaction times (RTs) should be faster on valid trials (when the target is embedded in the same shape as the prime) compared to invalid trials (when the prime’s shape contains a distractor).

### Experiment 1a

#### Method

##### Participants

Thirty undergraduates from Washington University in St. Louis participated for course credit. They all had normal or corrected-to-normal vision and normal color vision. The number of participants was selected to match that used by Weidler and Abrams (2016), who also studied the action effect. One participant was removed from the analysis because the conjoined accuracy (accuracy on both tasks) of this participant (81.3%) was more than 3 *SD* below the mean.

##### Stimuli, apparatus, and procedure

**Fig. 1:**
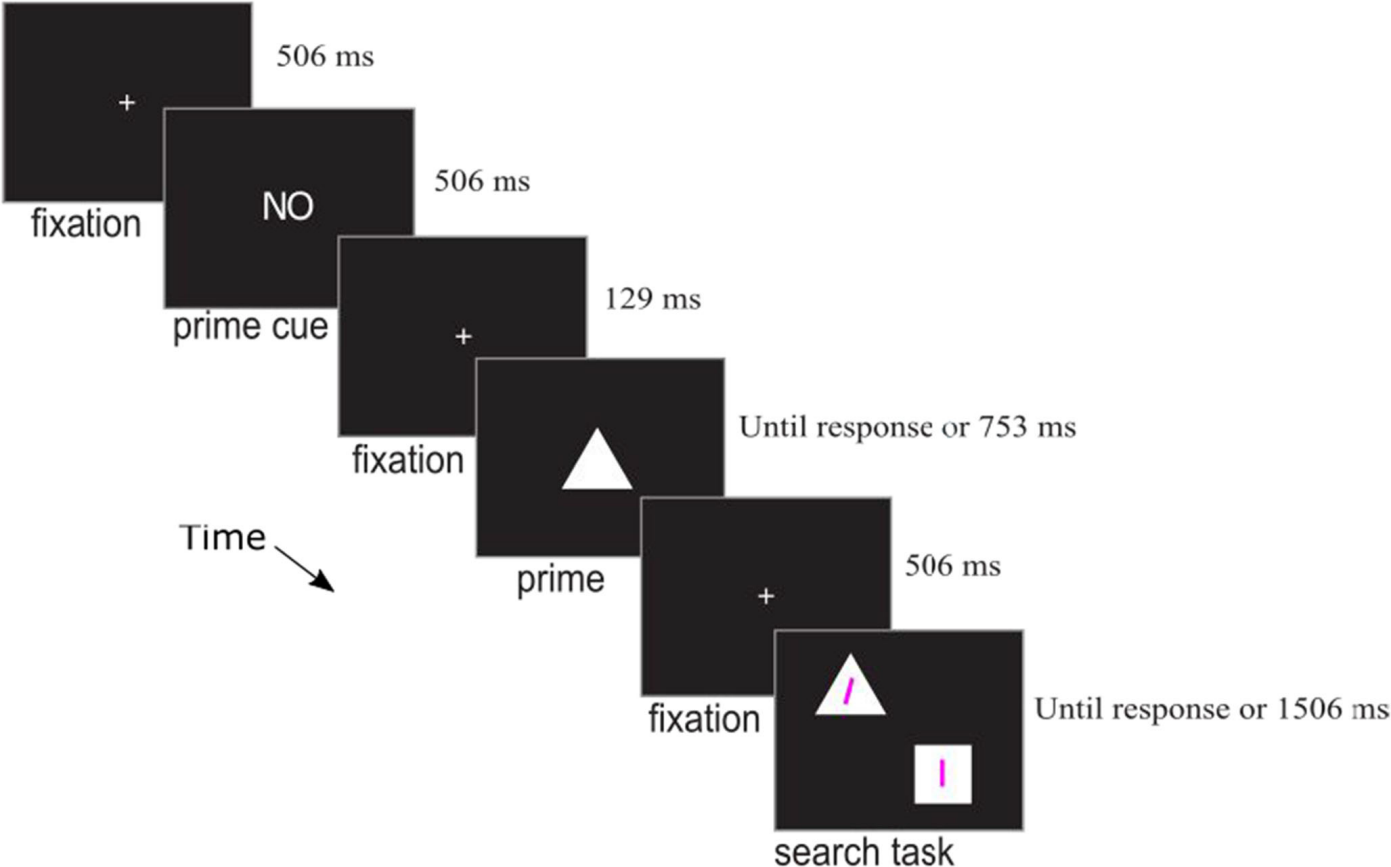
Sequence of events on a trial of Experiment 1a. This depicts an example of a *viewing* and *valid* trial

**Fig. 2:**
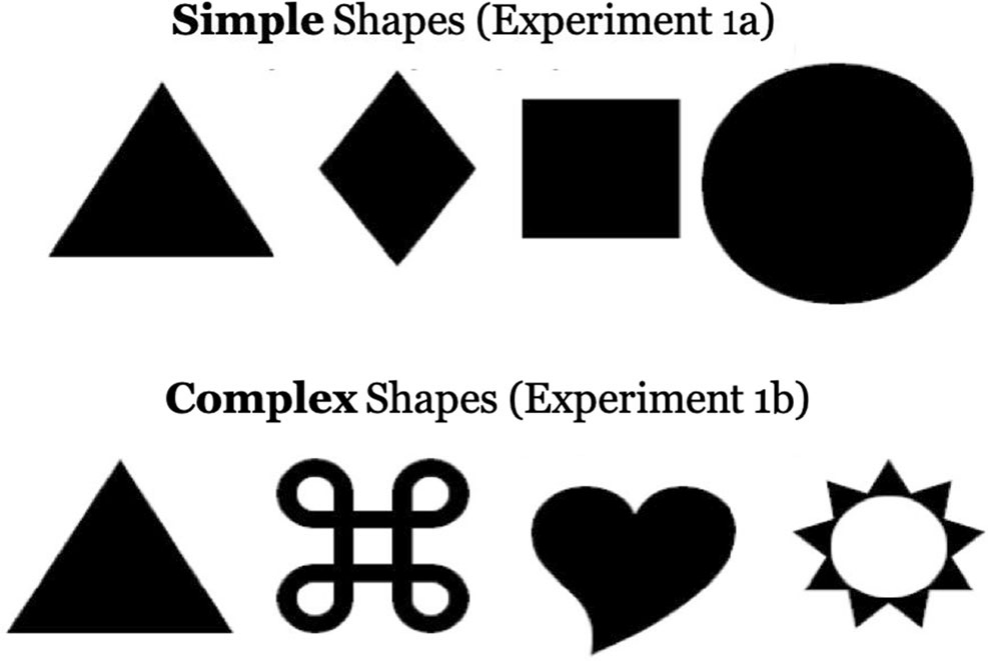
Stimuli used in Experiments 1a and 1b. In the experiments, the stimuli were white on a black background

Stimuli were presented on a CRT with an 85-Hz refresh rate. The sequence of events on a trial is shown in Fig. 1. Each trial began with a white fixation cross presented centrally for 506 ms (all stimuli were white and presented centrally unless otherwise noted). Next the word “GO” or “NO” appeared for 506 ms, followed by another fixation cross for 129 ms. The *prime* then appeared (a triangle, diamond, square, or circle, as shown in Fig. 2). If participants had previously seen the word “GO” they were instructed to press the space bar when the prime appeared (*action trials*). If participants had previously seen the word “NO” they were instructed to simply view the prime (*viewing trials*). The prime remained visible for 753 ms (or until response on action trials) and was followed by a 506-ms fixation period (with auditory feedback for an incorrect or too-slow trial).

Next, the search array appeared, containing two different shapes located at two corners of an invisible 16° square. Each shape was 7° high and contained a pink line (.12 × 2.6°). The target line was tilted 5° either to the left or right – participants’ task was to indicate its tilt by pressing the left or right arrow key – and the distractor line was vertical. The prime shape always appeared in the search display – on *valid* trials, it contained the tilted target line and on *invalid* trials it contained the vertical distractor line. The search array remained visible for 1,506 ms or until response and was followed by a 1,506-ms inter-trial-interval that contained 506 ms of auditory and visual feedback for trials that were incorrect or too slow.

##### Design

There were 48 unique trials presented in the experiment (2 action conditions × 2 validity conditions × 4 prime shapes × 3 distractor shape in the search display). Each of these trials was presented four times each for a total of 192 test trials, which were presented in a random order. The location of the target and distractor in the search array and the tilt of the target line were chosen randomly on each trial. Twenty-four practice trials preceded the test trials, and participants received breaks after every 48 test trials.

### Results and discussion

#### Action task

Participants responded correctly on 99.2% of action trials, and were slightly less accurate in action (*M* = 98.9%) than viewing (*M* = 99.5%) trials, *t*(28) = 1.88, *p* = .070. Participants’ average RT in the action task was 203 ms (*SD* = 68).

#### Search task

**Fig. 3:**
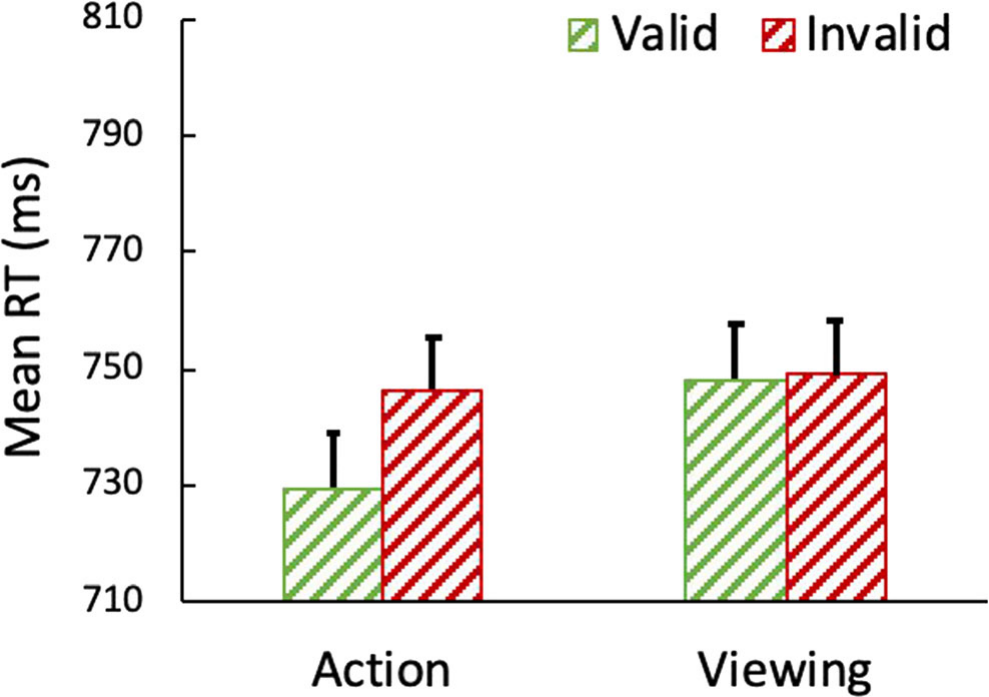
Reaction time (RT) for the search task in Experiment 1a. Error bars represent within-subject 95% confidence intervals

Conjoined accuracy (correct on both the action task and the search task) was high (96.8%; *SD* = 3.1%) and did not differ as a function of action, *F*(1,28) = 2.56, *p* = .120, or validity, nor did the two factors interact (*F*s < 1), so accuracy is not considered further. Reaction times (RTs) are shown in Fig. 3. A 2 action (action or viewing) × 2 validity (invalid or valid) within-subjects analysis of variance (ANOVA) was conducted on the RTs in the search task for trials in which both the action and search task were correct. There was a main effect of action *F*(1,28) = 4.30, *p =* .047, *η*^*2*^_*p*_ =. 13 (*M*_action_ *=* 738 ms, *M*_viewing_ = 749 ms) and a marginally reliable effect of validity, *F*(1,28) = 3.17, *p =* .086, (*M*_invalid_ *=* 748 ms, *M*_valid_ *=* 739 ms), η^2^_p_ =. 10. Additionally, there was a marginally reliable interaction between action and validity, *F*(1,28) = 3.78, *p =* .062, η^2^_p_ =. 12. Follow-up paired t-tests indicated that valid trials were reliably faster than invalid trials following an action, *t*(28) = 2.58, *p* = .016. (*M*_invalid_ *=* 746 ms, *M*_valid_ *=* 730 ms), but not following viewing the prime, *t* < 1 (*M*_invalid_ *=* 749 ms, *M*_valid_ *=* 748 ms).

In the current experiment, when all the stimuli shared a color and varied only in shape, there was limited evidence for the action effect. Participants were faster to find the search target when the previously acted-on shape contained the target than when it contained the distractor; however, the action-by-validity interaction that is indicative of an action effect was only marginally reliable. Therefore, these results imply that the previously reported effects of action (e.g., Buttaccio & Hahn, 2011; Weidler & Abrams 2014, 2016) may indeed be unique to color and may not extend to shape, limiting the generality of the effect. However, there may be an alternative explanation for the present results. Prior research has revealed that the perceptual system may be more sensitive to variations between simple colors (e.g., green and red) than between simple shapes (e.g., a square and circle; e.g., Theeuwes, 1992). Thus, the shapes used in the present experiment may have been too similar to each other – rendering our manipulation of shape only weakly effective. Experiment 1b explored this possibility.

### Experiment 1b

#### Method

##### Participants

Thirty new undergraduates from Washington University in St. Louis participated for course credit. One participant was excluded from the analysis due to conjoined accuracy more than 3 *SD* below the mean (that participant’s *M* = 51.0%).

##### Stimuli, apparatus, procedure, and design

The method was as in Experiment 1a with the exception that three of the stimulus shapes (all except the triangle) were different. Instead of the circle, diamond, and square in Experiment 1a, Experiment 1b presented three new figures (in addition to the triangle from Experiment 1a; see Fig. 2 for stimuli from both experiments). Although we did not formally assess the complexity and similarity of the shapes chosen, informal observations suggested that the items in the new set were subjectively more distinct from one another.

### Results and discussion

#### Action task

Participants responded correctly on 98.4% (*SD* = 2%) of trials, and again action trials (*M =* 97.5%) were less accurate than viewing trials (*M* = 99.3%) , *t*(28) = 3.02, *p* = .005. Average correct RT in the action task was 220 ms (SD = 63).

#### Search task

Conjoined accuracy for the remaining 29 participants was high (94.7%). A 2 action × 2 validity analysis revealed that participants were more accurate on viewing trials (*M* = 95.8%) than action trials (*M =* 93.7%), *F*(1,28) = 8.28, *p =* .008, η^2^_p_ =. 23. In addition, participants were more accurate on valid (*M* = 95.3%) than invalid (*M =* 94.2%) trials, *F*(1,28) = 4.40, *p =* .045, η^2^_p_ =. 14. Action and validity did not interact in the accuracy data, *F*<1.

**Fig. 4:**
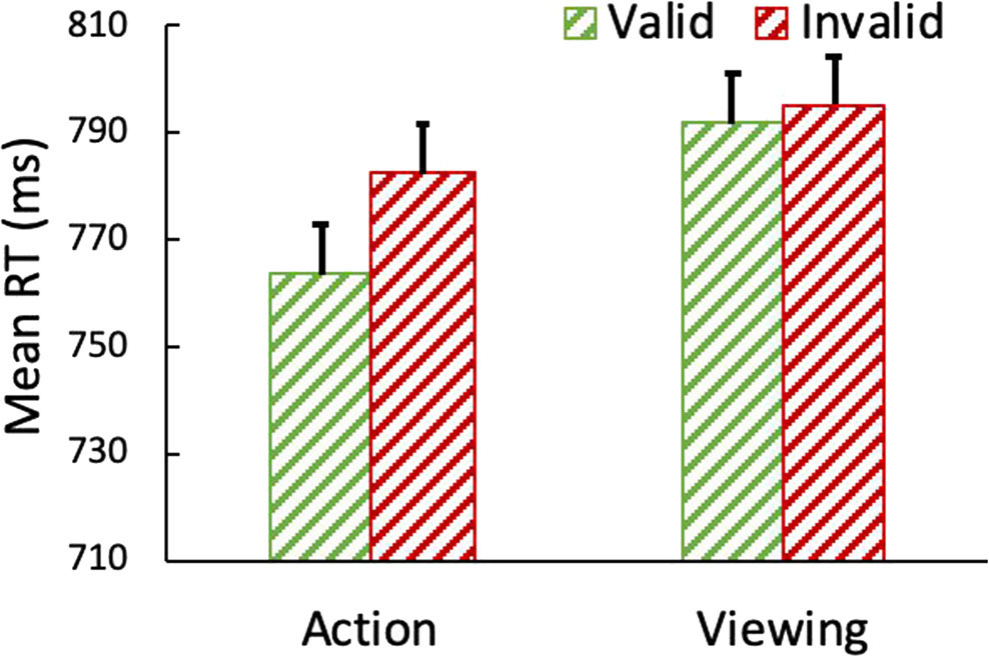
Reaction times (RTs) for the search task in Experiment 1b. After an action towards a shape (but not after mere viewing), participants were faster to find a target if it was contained within that shape (valid trials) than if that shape contained the distractor (invalid trials). Error bars represent within-subject 95% confidence intervals

Reaction times are shown in Fig. 4. The 2 action × 2 validity ANOVA on search RTs revealed that participants responded more quickly on action (*M* = 773) than viewing trials (*M* = 794), *F*(1,28) = 24.50, *p <* .001, η^2^_p_ =.47, and more quickly on valid (*M* = 778) than invalid (*M* = 789), trials, *F*(1,28) = 6.73, *p =* .015, η^2^_p_ =.19. Crucially, revealing the action effect, action and validity interacted, *F*(1,28) = 5.31 *p =* .029, η^2^_p_ =. 16. Following an action, participants were faster in valid than invalid trials, *t(*28) = 3.43, *p* = .002; however, the location of the prime shape relative to the target (i.e., validity) had no effect on viewing trials, *t* < 1.

In the present experiment, when the object shapes were highly visually distinct from each other, there was reliable evidence for the action effect. More specifically, following an action toward a prime shape, but not after merely viewing the shape, participants found the target more quickly if it was embedded in the shape from the action task than if that shape contained a distractor. Thus, this experiment provides the first evidence that simple action can affect perception of object features other than color.

## Experiments 2a, 2b, and 2c

The objects used in Experiments 1a and 1b varied only in shape, and by using highly distinct shapes, Experiment 1b revealed that an action can prioritize the acted upon shape – affecting the subsequent visual search. Nevertheless, like all previous studies (except one), it remains unclear whether action prioritizes the prime object and all of its features as a whole, or whether the features can be individually prioritized by the action. In Experiments 2a–2c, we investigated a series of experiments to address this question.

Additionally, in Experiments 2a–2c we changed the nature of the prime task so as to require participants to attend to the prime on all trials. In Experiments 1a and 1b it might have been possible for participants to ignore the prime on some trials on which an action was not required. If that had happened, the results we observed might reflect differential allocation of attention to the prime, and not necessarily the consequences of an action. With the changes to the prime task here, that would not have been possible in Experiments 2a–2c.

### Experiment 2a

#### Method

##### Participants

Twenty-four students from Tsinghua University participated in the experiment. All participants reported having normal or corrected-to-normal vision. The number of participants was selected to match that used by Weidler and Abrams (2014), and differs from that used in Experiments 1 because this experiment was conducted in a different lab.

##### Stimuli, apparatus, and procedure

**Fig. 5:**
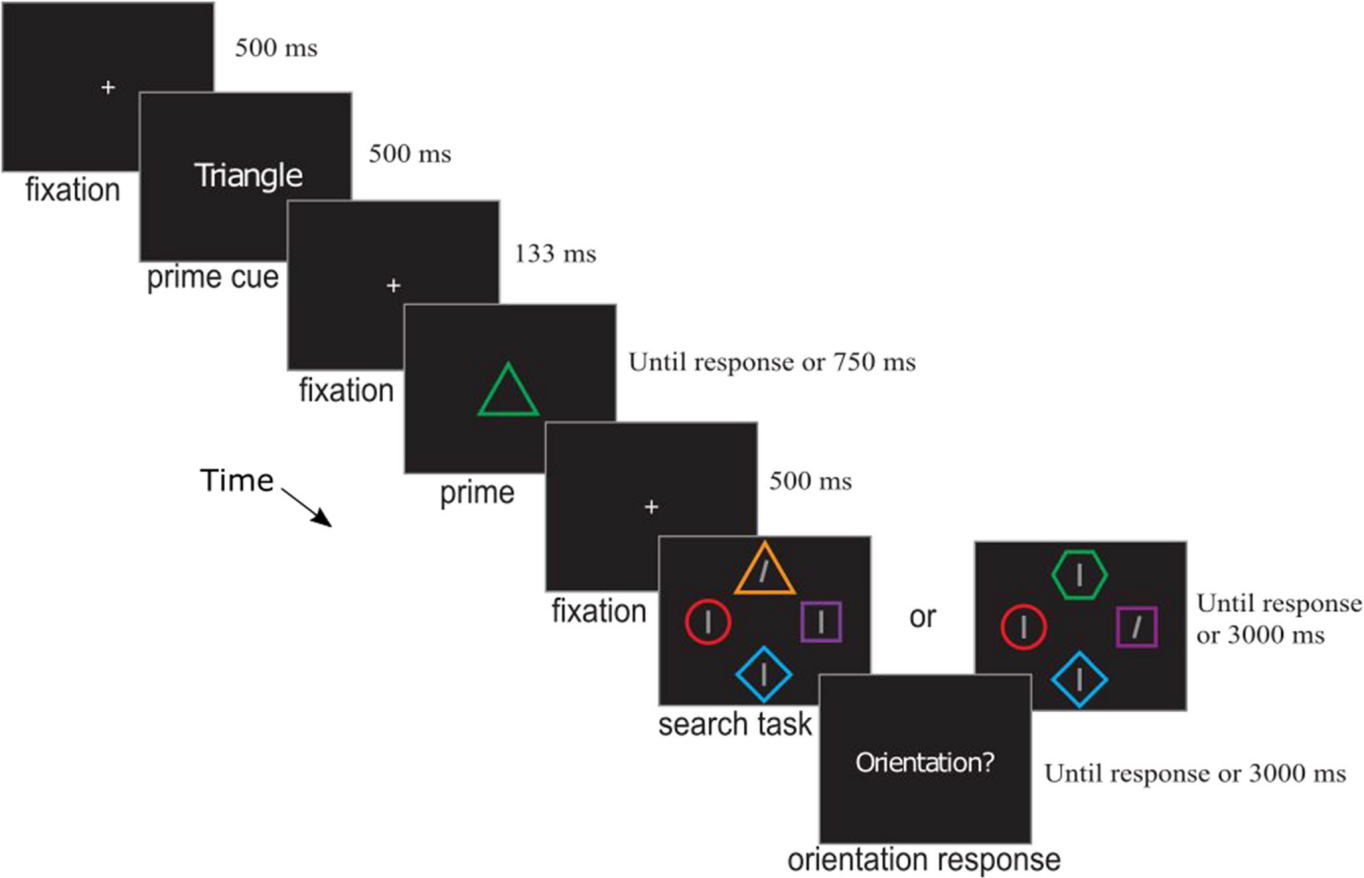
Sequence of events of Experiment 2a on a shape-valid trial (the “search task” example on the left) and a color-invalid trial (the example on the right). See text for additional explanation. Images are not drawn to scale

Stimuli were presented on an LED screen with a resolution of 1,920 × 1,080 at 60 Hz with Psychtoolbox 3.0 (Brainard, 1997; Kleiner et al., 2007) running under a Matlab R2016a environment (Mathworks, Natick, MA, USA). The sequence of events in the experiment is illustrated in Fig. 5.

The experiment was very similar to Experiments 1a and 1b. Participants first performed an action task and then a subsequent visual search task. On each trial, participants were first presented a 2° white fixation cross at the center of a black screen for 500 ms. Next, a shape name (3.9°) randomly chosen from six shape names (circle, square, triangle, diamond, hexagon, and pentagon) was displayed at the center of the screen for 500 ms, followed by a fixation cross for 133 ms. Next, a colored object (the prime) with a size of 4-deg^2^ appeared, with its color chosen from six colors: blue, RGB = [0,0,255]; green, RGB = [0,128,0]; orange, RGB = [255,97,0]; purple, RGB = [102,0,102]; red, RGB = [255,0,0]; or yellow, RGB = [204,204,0]; and its shape chosen from the six shapes corresponding to the six shape names. Participants were instructed to press the spacebar using their left hand as quickly as possible if the shape of the object matched the previously presented shape name (*action trials*). If they mismatched, participants were instructed not to respond (*viewing trials*).

After participants responded or 750 ms, another fixation cross was presented at the screen center for 500 ms, followed by a search display in which four 4-deg^2^ colored objects selected from the same six possible colors and shapes were presented. They were presented at four of eight possible positions on an imaginary circle around the screen center with a radius of 6.43°. Three of these objects contained a vertical grey (RGB = [128,128,128]) line 1.13° in length and 0.12° in width, whereas the fourth object contained a tilted line (tilted angle of -5° or +5°) of the same size. Participants were instructed to press the up-arrow key using their right hand as soon as they saw the tilted line. After 3,000 ms or after participants responded, the search display disappeared and the word “Orientation?” was shown to indicate that participants should press the left or right arrow key depending on the orientation of the tilted target. Participants had 3,000 ms to respond to this question and then the next trial began immediately after the orientation response.

There were two critical types of search displays. In the *color condition*, one of the objects shared its color with the previously presented prime, and the other objects all had different colors and shapes both from the prime and each other. In the *shape condition*, one of the objects shared its shape with the prime, and the other objects all had different shapes and colors both from the prime and each other. Note that in the color condition, the prime shape was never presented in the search display and in the shape condition the prime color was not in the search display. We also included filler trials in which neither the prime shape nor color appeared in the search display.

Validity was manipulated in the color and shape trials. On *valid trials*, the target object possessed a feature (either color or shape) that matched the prime whereas on *invalid trials*, one of the distractor objects possessed the prime-matching feature.

##### Design

The experiment employed a 2 (action task: action vs. viewing) × 2 (shared feature: color vs. shape) × 2 (validity: valid vs. invalid) within-subject design. There were four possible invalid trial types (action task: action vs. viewing × shared feature: color vs. shape), which were each presented 80 times for a total of 320 invalid trials. The four possible valid trial types (action task: action vs. viewing × shared feature: color vs. shape) each occurred 40 times, for a total of 160 valid trials. Finally, 40 action filler trials and 40 viewing filler trials were also included. All 560 trials were mixed together and presented in a random order.^1^ The colors and shapes used during each trial were selected randomly subject to constraints required by the condition. For example, the shape of the prime matched the shape name (prime cue) only on action trials, and in the color condition one of the elements in the search array shared its color with that of the prime, with that object containing the target only on valid trials. The location of the target and distractors in the search array and the orientation of the target line were also chosen randomly on each trial. Prior to the test trials, 20 practice trials were performed. During the experiment, participants could choose to take a rest after each 112 trials.

#### Results and discussion

##### Action task

Participants responded correctly on 97.4% (*SD* = 1.0%) of trials, and again action trials (*M =* 96.5%) were less accurate than viewing trials (*M* = 98.4%), *t*(23) = 2.94, *p* = .007. Average correct RT in the action task was 440 ms (*SD* = 40).

##### Search task

Conjoined accuracy was 96.4%. Given that participants responded correctly in most of the trials, the accuracy data was not further analyzed.

For the RT results we analyzed the latency to indicate that the search target had been located. Only trials with correct responses in both tasks were included. RTs faster than 200 ms or slower than 2,500 ms were excluded, resulting in an exclusion of 0.3% of the total trials.

**Fig. 6:**
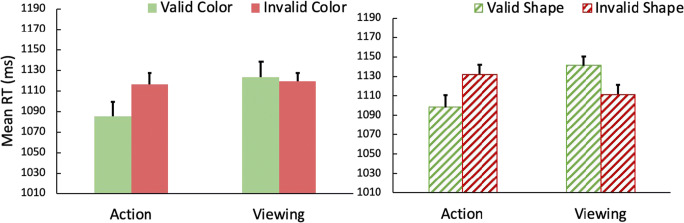
Mean reaction times (RTs) in the visual search task of Experiment 2a, in which action toward the prime required a shape match. Under both the color condition (**left panel**) and the shape condition (**right panel**), an action effect was found: participants responded faster on valid trials than invalid trials but only after an action had been made. On viewing trials, no significant validity effect was found. Error bars represent within-subject 95% confidence intervals

RTs are shown in Fig. 6. A 2 (action task: action vs. viewing) × 2 (shared feature: color vs. shape) × 2 (validity: valid vs. invalid) within-subjects ANOVA was conducted on the mean RT. There was a marginally significant main effect of action, *F*(1,23) = 3.88, *p* = .061, *η*^*2*^_*p*_ = .14. Participants responded slightly faster in the search task when they previously made an action in the prime task compared to only viewing. There was an overall action effect, revealed by a significant interaction between action and validity, *F*(1,23) = 6.16, *p* = .021, *η*^*2*^_*p*_ = .21. Post hoc tests revealed that on action trials, participants responded significantly faster on valid (*M* = 1,090) versus invalid (*M* = 1,124) trials, *t*(23) = 2.54, *p* = .018, but on viewing trials RTs were equivalent on valid (*M* = 1,132) and invalid (*M =* 1,115) trials, *t*(23) = 1.63, *p* = .116. The three-way interaction of the three factors was not significant, *F*(1,23) < 1, indicating that the action effect was equivalent when the target shared either the color of the prime or the shape of the prime. All other two-way interactions and main effects were not significant, *Fs* < 1.73, *ps* > .2.

In the present experiment we found that an action toward an object prioritized the color or shape of that object in a subsequent search, even when the target of the search only either matched the color or the shape of the prime object. The results not only replicate previous findings of action prioritizing the color of the prime (e.g., Buttaccio & Hahn, 2011) and our findings in Experiment 1b, which showed that action can prioritize the shape, but also further reveal that a simple action is capable of enhancing search for individual visual features of the acted-on object and the benefit of an action is not limited to the specific combination of features contained in the prime.

One aspect of the present experiment deserves further scrutiny. Participants were specifically instructed to attend to the shape of the prime because an action was to be made only if the prime shape matched the shape word that had been presented earlier. Though using this task can avoid the potential weakness of Experiments 1a and 1b, in which participants might pre-determine their attentional state before the onset of the prime stimulus based on the cue, and though such instructions cannot account for the fact that action to the prime also prioritized the prime’s color, they might account in part for the effect of action on the shape of the prime. Thus, it is of interest to determine whether an action effect for both shape and color can also occur in the absence of explicit direction to attend to shape. That was the purpose of Experiment 2b.

### Experiment 2b

The results of Experiments 2a showed that action can prioritize search individually for either the color or the shape of the acted-on object (the prime). However, the cue word in Experiment 2a explicitly directed participants to attend to the shape of the prime leaving unanswered the question of whether such a direction played a role in the findings. To investigate this, here we changed the cue word from a shape name to a color name.

#### Method

##### Participants

Twenty-four students from Tsinghua University participated in the experiment. All participants reported having normal or corrected-to-normal vision and had not participated in the earlier experiments.

##### Apparatus, stimuli, and procedure

The method was similar to Experiment 2a except for the following: participants were first shown a color word selected from the same color set used in Experiment 2a, and they were instructed to make an action (i.e., press a key) if the color of the prime object matched the color word. Second, we increased the number of filler trials so that each session contained 160 valid, 320 invalid, and 160 filler trials yielding a total of 640 trials in the session. Participants were provided a rest after each 80 trials.

#### Results and discussion

##### Action task

Participants responded correctly on 98.4% (*SD* = 1.4%) of trials, and again action trials (*M =* 98.1%) were less accurate than viewing trials (*M* = 98.8%) , *t*(23) = 2.17, *p* = .041. Average correct RT in the action task was 413 ms (*SD* = 40).

##### Search task

Conjoined accuracy was 97.2%. Incorrect responses and trials with RTs shorter than 200 ms or longer than 2,500 ms were excluded, resulting in removal of 3.14% of the total trials.

**Fig. 7:**
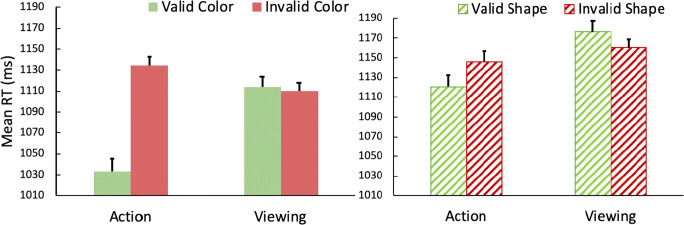
Mean reaction times (RTs) in the visual search task primed by the color word, from Experiment 2b. An action effect was found for color (**left panel,** color condition) but not shape (**right panel,** shape condition). Error bars represent within-subject 95% confidence intervals

Reaction times are shown in Fig. 7. We performed a 2 (prime task: action vs. viewing) × 2 (shared feature: color vs. shape) × 2 (prime feature validity in visual search task: valid vs. invalid) within-subject analysis of variance (ANOVA) on the remaining RTs. The main effect of action was significant, *F*(1,23) = 6.36, *p* = .019, *η*^*2*^_*p*_ = .22, with participants responding faster on action compared to viewing trials. The main effect of validity in the search task was also significant, *F*(1,23) = 10.79, *p* = .003, *η*^*2*^_*p*_ = .32, with shorter RTs on valid compared to invalid trials. The main effect of shared feature was not significant, *F*(1,23) = 2.93, *p* = .101.

The analysis also yielded a significant three-way interaction among the three factors, *F*(1,23) = 10.78, *p* = .003, *η*^*2*^_*p*_ = 0.32. Thus, separate 2 × 2 ANOVAs were conducted on the color and shape conditions. The three-way interaction occurred because there was an action effect (i.e., an interaction between prime task and prime feature validity) when the shared feature was color, *F*(1,23) = 29.55, *p* < .001, *η*^*2*^_*p*_ = 0.56, but not when the shared feature was shape, *F*(1,23) < 1. Post hoc tests further showed that, under the color condition, participants responded significantly faster on valid trials (*M* = 1,033) than invalid trials (*M* = 1,134) only after an action, *t*(23) = 6.80, *p* < .001, but not after viewing, *t*(23) < 1, with mean RTs 1,114 and 1,110 ms, respectively, for the valid and invalid conditions. Under the shape condition, RTs in the search task on valid trials and invalid trials did not differ regardless of whether participants had made an action (valid, *M =* 1,106; invalid, *M =* 1,103) or just passively viewed the display (valid, *M =* 1,123, invalid, *M =* 1,116) in the prime task, *ts* < 1.

In this experiment, an action effect was found for color, partially replicating the results in Experiment 2a that action can prioritize individual visual features of the acted-on object in a subsequent visual search task. However, we did not find an action effect for shape here. That is, an action did not lead to an advantage in search when the shape of the prime matched that of the target, differing from the results of Experiment 2a. In that experiment attention was explicitly directed to shape, yet there is evidence that color was processed also (because there was an action effect for color). That may be because color is highly salient, and the different colors used may be very distinct from one another (and there are multiple other reports of color-based action effects when it is task-relevant; e.g., Huffman & Pratt, 2017; Weidler & Abrams, 2014; 2016; Weidler et al., 2018). The shapes used here, on the other hand, may be less distinct, and were not explicitly directed to be attended, perhaps explaining why shape did not produce an action effect in the present experiment.

The present results also contrast somewhat with those from Experiment 1b in which we also found an action effect for shape. Those results were obtained despite the fact that attention was not explicitly drawn to object shape in that experiment either. However, the stimuli in Experiment 1b were all white – they varied only in shape, not in color, and as a result shape was the only feature that distinguished one object from another. And the shapes there were selected to be highly distinct – unlike those used in the present experiment. Additionally, the search elements in Experiment 1b were exact matches to the primes used there.

Thus, it still remains an open question if shape will yield an action effect when the shapes during search are not combined with the same color as the prime and when attention is not explicitly drawn to either shape or color. To test this, in the next experiment, we used a modification that does not require participants to attend explicitly to either shape or color.

### Experiment 2c

In the present experiment we examined whether an action will enhance subsequent visual search for the individual visual features of the prime when the prime’s features are completely irrelevant in the prime task.

#### Method

##### Participants

Twenty-four students from Tsinghua University participated in the experiment. All participants reported to have normal or corrected-to-normal vision and did not participate in the earlier experiments.

##### Apparatus, stimuli, and procedure

Experiment 2c was identical to Experiment 2b except for the prime task. No words were used in the prime task. Instead, participants were instructed to make a manual response if nothing was superimposed on the prime, but to withhold the action when a white “X” (1°high) was superimposed on the prime (on half of the trials). The instructions for the priming phase were: “Please always press the space bar as soon as you see a shape, unless there is an ‘X’ inside the shape”.

#### Results and discussion

##### Action task

Three participants’ data were excluded because their conjoined accuracies were more than three standard deviations below the mean (83.9%, 90.8%, and 91.9%, respectively). The remaining participants responded correctly on 98.7% (*SD* = 0.6%) of trials in the action task, and again action trials (*M* = 98.3%) were slightly less accurate than viewing trials (*M* = 99.0%) *, t*(23) =1.89*, p* =.074. Average correct RT in the action task was 423 ms (*SD* = 34).

##### Search task

Conjoined accuracy was 97.9% (*SD* = 0.9%) for the 21 remaining participants. When analyzing the RTs, incorrect responses as well as RTs that were shorter than 200 ms or longer than 2,500 ms were also excluded, which in total resulted in an exclusion of 2.75% of the total trials.

**Fig. 8:**
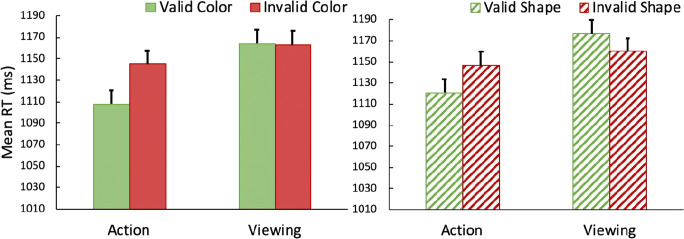
Mean reaction times (RTs) in the visual search task from Experiment 2c. As in Experiment 2a, a significant action effect was observed for both color (**left panel**) and shape (**right panel**). Error bars represent within-subject 95% confidence intervals

RTs are shown in Fig. 8. A 2 action × 2 shared feature× 2 validity ANOVA revealed that there was a significant main effect of action, *F*(1,21) = 15.70, *p* = .001, *η*^*2*^_*p*_ = .44, with participants responding faster overall in the search after an action toward the prime, compared to viewing. As in the earlier experiments an action effect was observed, with a significant two-way interaction between prime task and validity, *F*(1,21) = 6.89, *p* = .016, *η*^*2*^_*p*_ =.26. Simple main effect analysis showed that following an action participants were faster to search for the target on valid trials (*M* = 1,114) compared to invalid trials (*M* = 1,146), *t*(23) = 2.80, *p* = .011. If participants just viewed the prime without making an action, however, no difference was found between valid trials (*M* = 1,170) and invalid trials (*M* = 1,161), *t*(23) < 1. Importantly, differing from Experiment 2b, the three-way interaction was not significant, *F*(1,21) < 1, indicating that the benefit of action was equivalent for color and shape, here in the absence of an explicit cue to either color or shape. All other main effects and two-way interactions were not significant, *Fs* < 2.1, *ps* > .16.

In the present experiment, there was an action effect for both shape and color. That is, participants were faster to find the search target when it was embedded in an object that matched either the color or the shape of the prime – but only after they had made an action to the prime. And that result was obtained with search stimuli that matched only the color or shape of the prime, but not both, implying that the action is capable of prioritizing the features of acted-on objects. Importantly, in the present experiment attention was not explicitly directed toward either color or shape (of the prime or of the search elements) – and there was no need for participants to process either color or shape to perform the prime task. Nevertheless, both the color and shape of the acted-on stimulus affected subsequent search. These results extend the findings of Experiment 1b, in which an action effect was also observed for shape where shape was the only dimension on which the stimuli differed, and the search elements were exact matches to the prime. Results from this experiment also extend those from Experiment 2a, in which an action effect was found for both color and shape, yet in which it had been necessary for participants to process the shape of the prime explicitly.

## General discussion

In the present study, we explored the action effect: the influence of a simple action on the subsequent allocation of attention toward features of the acted-on object (the prime). Previous investigations have examined only whether the color of the prime is prioritized after an action, leaving unknown whether other visual features can also be affected. Additionally, prior research has used search elements that were exact matches to all features of the prime – raising the question of whether action can prioritize individual visual features as opposed to specific combinations of features in an integrated object representation. Our set of experiments presents an initial answer to both of these questions.

Experiments 1a (marginally) and 1b showed that action can prioritize object shapes. In those experiments, shape was the only dimension on which the stimuli varied, and the search elements were exact matches to the prime. These findings are the first to extend the action effect to visual features other than color.

In Experiments 2a–2c, we investigated whether action can influence subsequent attention to individual features of the acted-on objects. In those experiments, the search elements were never an exact match to the prime. Instead, they sometimes matched either the color (but not the shape) or the shape (but not the color) of the prime. In Experiment 2a, when participants made an action based on the prime’s shape, there was an action effect when the search target matched the color of the prime (and not the shape) or the shape of the prime (and not the color), revealing that an action can prioritize visual features. In Experiment 2b, when participants’ decision to act required evaluating the prime’s color, we replicated an action effect for color, but not for shape. Finally, in Experiment 2c, when neither the prime’s color dictated the action, we found an action effect for both shape and color features when they appeared in a search object that was not an exact match to the prime.

### Differences between color and shape

Our results revealed some differences between the features of shape and color. Color yielded an action effect both when the color of the prime was explicitly attended (Experiment 2a) and when it was not necessary for participants to attend to color (Experiments 2b and 2c). Shape, on the other hand, yielded an action effect only when color was not explicitly attended: Experiments 1a and 1b used stimuli that varied only on shape, and Experiments 2a and 2c did not require attention to color. These results are consistent with other findings showing that color yields strong perceptual effects, perhaps because color (and color differences) are highly salient (Goolsby & Suzuki, 2001).

### Relation to theory-of-event coding

Our finding that the component features of an object are prioritized by an action provides details about the way in which actions can interact with the visual representations of objects. The theory-of-event coding proposes that actions are integrated together with the representations of acted-on objects in working memory: an extensive line of research has provided evidence that object features and action features (or action codes) can be bound together into representations referred to as event files (Hommel, 1998, 2004). Importantly, it has been shown that individual object features, not conjunctions of features, are bound to the actions (e.g., Hommel & Colzato, 2004; Hommel, 2007). And indeed, our results also suggest that individual features, such as color alone or shape alone, can be prioritized by a recently performed action, raising the possibility that some of the same mechanisms may be involved in the action effect and in event file representations. Additionally, recent converging evidence from psychophysical and neurological experiments suggests that visual working memory and action are related in not just a hierarchical feedforward manner, but rather by concurrent networks in which visual and motor working memory interact with each other (van Ede et al., 2019; van Ede, 2020). These sorts of connections may be what permits a recently performed action to affect subsequent perception for a brief period of time.

Research on event files may also help explain why shape appears to have been prioritized by action to a somewhat lesser degree than color. When attention was directed to shape (Experiment 2a) and when attention was not directed to either color or shape (Experiment 2c), we found an action effect for shape. But when attention was directed to color (Experiment 2b), we did not find such an effect. Singh et al. (2018), discussed earlier, showed that an irrelevant feature of an object might not be bound to a response (i.e., incorporated into an event file) if it was not attended. Those findings imply that shape might be prioritized by action without an explicit attentional direction (Experiment 2c), or when attention is directed to shape (Experiment 2a), but color yields an action effect, perhaps because of its salience, regardless of the attentional direction (i.e., in Experiments 2a–2c).

Differences in the action effect for color and shape may also be understood based on some findings from studies of priming. Kristjánsson (2006) argued that a task-irrelevant feature may not be subject to priming if its neural substrate overlaps with one of the task relevant features. In our experiments, line orientation (i.e., shape) was the relevant feature in the search task. Following Kristjánsson’s logic, we might not expect robust priming for the shape of the prime, but would expect robust priming for its color, consistent with the pattern we reported.

### Relation to biased competition

Our findings are also consistent with an explanation that suggests that action exerts its effect on visual search by biasing the competition for attentional representation in favor of objects that match the acted-on object (Huffman & Pratt, 2017). According to that explanation, action enhances vision via some of the same mechanisms through which attention can have a similar effect. In particular, it’s known that attention to an object results in biased neural activity that favors the object’s features in brain regions that process those features, such as areas V2 and V4 (Reynolds, et al., 1999) – areas in which the neurons respond to features such as the orientation or the color of an object (Anzai, Peng, & Van Essen, 2007; Motter, 1994; as well as to combinations of such features, Hegdé & Van Essen, 2000). Additionally, it has been shown that attention to stimulus orientation in a specific location can enhance orientation-specific responses in V4 even for stimuli in unattended locations (McAdams & Maunsell, 2000). And, attention to a feature such as color has been shown to heighten brain responses to the attended color throughout the visual field (Saenz, Buracas, & Boynton, 2002). These latter findings suggest a way in which action toward a prime object could enhance search throughout the visual field for elements matching either the color or shape of the prime (but differing on the other dimension).

### Implications for exogenous feature-based attention

The present results may contribute also to our understanding of *exogenous* feature-based attention, that is, feature-based prioritization caused not by salience or by an individual’s top-down goals. In our case, the enhancement of features of the prime was produced by the mere requirement to make an action in response to it. Importantly, the features of the prime that were prioritized (color and shape) were irrelevant to the task. While our results support the existence of exogenous feature-based attention, a recent study has failed to find support for it (Donovan et al., 2020). One possible explanation for the discrepancy is that action helps to boost the response over and above the effect of merely presenting a stimulus. Indeed, that is the very definition of the action effect.

### Conclusions

Performing efficient actions is an important part of everyday behavior. The present experiments have shown that even simple actions can have a profound effect on subsequent perception, prioritizing attentional selection of objects that share only basic features with the acted-on object. Such a bias may stem from the fact that ongoing (and especially repetitive) actions may be likely to share their target features with those of earlier actions, such as when one is picking berries, or engaged in an intense session of whack-a-mole.

## Footnotes

1 Note that the prime was a valid predictor of the target location on 29% of trials – slightly more than the 25% expected by chance. However, while that might lead to an overall validity effect, it was not confounded with the action task, and as a result it does not compromise our ability to address the questions of interest. Indeed, it is an interesting question to consider whether an action might prioritize search when the search includes a strong top-down component, although the present experiments have not been designed to address that. An action effect has been shown to occur for pop-out searches (in which the target is highly salient; Weidler & Abrams, 2016), and, as is typical, in searches in which the target does not differ from distractors on the basis of either top-down or bottom-up factors (e.g., Weidler & Abrams, 2014).
